# Mechanisms of Resistance to Anti-PD-1 Immunotherapy in Melanoma and Strategies to Overcome It

**DOI:** 10.3390/biom15020269

**Published:** 2025-02-12

**Authors:** Magdalena K. Zielińska, Magdalena Ciążyńska, Dorota Sulejczak, Piotr Rutkowski, Anna M. Czarnecka

**Affiliations:** 1 Department of Soft Tissue/Bone Sarcoma and Melanoma, Maria Sklodowska-Curie National Research Institute of Oncology, 02-781 Warsaw, Poland; magda.zielinska20@gmail.com (M.K.Z.); piotr.rutkowski@pib-nio.pl (P.R.); 2 Faculty of Medicine, Warsaw Medical University, 02-091 Warsaw, Poland; 3 Chemotherapy Unit and Day Chemotherapy Ward, Specialised Oncology Hospital, 97-200 Tomaszów Mazowiecki, Poland; magdalena.ciazynska@nu-med.pl; 4 Department of Dermatology, Paediatric Dermatology and Oncology Clinic, Medical University of Lodz, 91-347 Łódź, Poland; 5 Department of Experimental Pharmacology, Mossakowski Medical Research Institute, Polish Academy of Sciences, 02-106 Warsaw, Poland; dots@op.pl

**Keywords:** melanoma, immunotherapy, nivolumab, pembrolizumab, primary resistance, secondary resistance, innate anti-PD-1 resistance signature (IPRES), melanoma immune evasion, anti-PD-1, anti-PD-1 therapy resistance, checkpoint inhibitors

## Abstract

Resistance to anti-PD-1 therapy in melanoma remains a major obstacle in achieving effective and durable treatment outcomes, highlighting the need to understand and address the underlying mechanisms. The first key factor is innate anti-PD-1 resistance signature (IPRES), an expression of a group of genes associated with tumor plasticity and immune evasion. IPRES promotes epithelial-to-mesenchymal transition (EMT), increasing melanoma cells’ invasiveness and survival. Overexpressed AXL, TWIST2, and WNT5a induce phenotypic changes. The upregulation of pro-inflammatory cytokines frequently coincides with EMT-related changes, further promoting a resistant and aggressive tumor phenotype. Inflamed tumor microenvironment may also drive the expression of resistance. The complexity of immune resistance development suggests that combination therapies are necessary to overcome it. Furthermore, targeting epigenetic regulation and exploring novel approaches such as miR-146a modulation may provide new strategies to counter resistance in melanoma.

## 1. Introduction

The implementation of immunotherapy in clinical practice has marked a significant advancement in cancer treatment. Immunotherapy enables targeting of checkpoints that regulate T-cell activity, which represents a vital component of the immune response against cancers. The interaction between programmed cell death 1 (PD-1) and its ligands PD-L1 and PD-L2 is critical for promoting the escape of cancer cells from immune surveillance. Monoclonal antibodies targeting PD-1, namely pembrolizumab and nivolumab, are used in melanoma treatment. FDA approved these for treating nonresectable or metastatic melanoma in 2014 [1]. Nivolumab and pembrolizumab belong to the IgG4 subclass and demonstrate strong affinity and specificity for PD-1 [2]. In clinical practice, some patients respond to treatment, but a subset of patients present with primary resistance (i.e., do not respond initially to immunotherapy) or develop secondary resistance (i.e., recurrence occurs after periods of extended disease control, such as a partial or complete response followed by progressive disease, according to RECIST 1.1). A large group of patients develops resistance to treatment within three months to three years of anti-PD-1 therapy [1] (Table 1). Additionally, primary (innate) and secondary (acquired) resistance [3,4,5,6], an intermediate phenotype known as adaptive resistance has also been recognized by some authors [7]. At the cellular level, resistance to immunotherapy covers mechanisms that prevent any initial response to treatment and mechanisms that develop later, leading to tumor escape and progression despite the initial therapeutic benefit [3,4,5,6].

The initial efficacy of checkpoint inhibitors has been demonstrated in approximately 45–65% of patients with melanoma in the first line of treatment [8,9]. Nevertheless, over 40% of patients do not respond initially to immunotherapy due to pre-existing primary resistance mechanisms, thus categorizing them as primary non-responders. Furthermore, 25–30% of patients with metastatic melanoma who initially respond to treatment eventually experience relapse due to secondary resistance [10]. Consequently, only a subset of patients experience long-term survival benefits [11]. For instance, in the KEYNOTE-001 trial assessing the efficacy of pembrolizumab, the five-year overall survival (OS) rate was 34% for all patients and 41% for those who had not previously undergone treatment, while the median overall survival (mOS) was 23.8 months and 38.6 months, respectively [12]. At ten years, the OS rate was 34.0% for all patients [13]. Approximately 25% of patients who initially responded to treatment exhibited disease progression within a median period of 21 months [8]. Among the 655 patients who received treatment, 164 (25%) experienced progressive disease (PD) as the best response, so presented with primary resistance. In the cohort of patients who received first-line treatment, there were 32 patients (21%) with primary resistance. In the CheckMate 067 trial, the median overall survival (mOS) was 72.1 months for patients treated with the combination of nivolumab and ipilimumab, 36.9 months for those who received nivolumab alone, and 19.9 months for monotherapy with ipilimumab [12]. A study by Amaral et al. (2020) revealed that among 319 patients with stage IV melanoma, 40% exhibited primary resistance to checkpoint inhibitors. Of the 319 patients, 145 were treated with nivolumab + ipilimumab, 128 with pembrolizumab, and 46 with nivolumab. During a median follow-up period of 22 months, patients with primary resistance exhibited significantly lower overall survival rates at 1, 2, and 3 years (41%, 15%, and 10%, respectively) compared to patients who achieved disease control (91%, 81%, and 65%) [14]. A study conducted by Hepner et al. (2024) involving 299 patients with melanoma provided insights into the clinical pattern of acquired resistance to PD-1 inhibitors. Most patients (65%) exhibited progression at a single organ site, with the most common sites being the lymph nodes (38%), brain (25%), and lungs (22%). Overall survival after axillary recurrence was correlated with solitary progression, which subsequently resulted in better outcomes. Notably, despite the development of resistance with the continued administration of immunotherapy, it resulted in a median overall survival of more than three years, indicating the potential for sustained benefits in a subset of patients [15,16].

The molecular mechanisms underlying resistance to immunotherapy involve interactions between the host and tumor within the tumor microenvironment. Multiple mechanisms overlap between primary and secondary resistance [14]. Various intracellular, intratumoral, and systemic processes, including molecular alterations, cellular dynamics, metabolic adaptations, and microbiome effects, contribute to evading ICI-enhanced immunosurveillance in melanomas [17]. In this review we aim to describe primary and secondary mechanisms of anti-PD-1 resistance in patients with melanoma and summarize existing and potential therapeutic strategies, including combination therapies and epigenetic modulation approaches.

## 2. PD-1 and PD-L1

PD-1 (CD279) is a receptor protein found on the surface of many cell types [2]. PD-1 is a significant regulator of immune suppression and a promoter of self-tolerance by modulating T-cell activity. PD-1 is expressed in activated CD8+ T cells, B cells, macrophages, dendritic cells (DC), monocytes, and natural killer cells, particularly after prolonged exposure to cancer antigens [18]. PD-1 can bind to two B7 family ligands, PD-L1 (B7-H1, CD274) and PD-L2 (B7-DC, CD273), which share 37% sequence similarity and originate through gene duplication [19,20,21]. This interaction suppresses the activity of activated peripheral T lymphocytes, thereby attenuating the immune response against tumor cells, particularly by reducing the ability of T cells to divide and respond [2,22]. PD-L1 is a transmembrane protein and a co-inhibitory factor in the immune response. It binds to PD-1, leading to the reduced proliferation of PD-1-positive cells and cytokine secretion suppression. PD-L1 expression is commonly observed in macrophages, specific activated T cells and B cells, dendritic cells (DC), and specific epithelial cells, particularly under inflammatory conditions such as those induced by interferons (IFNs) [23]. Additionally, tumor cells use PD-L1 expression as an “adaptive immune mechanism” to evade anti-tumor responses [24].

**Figure 1:**
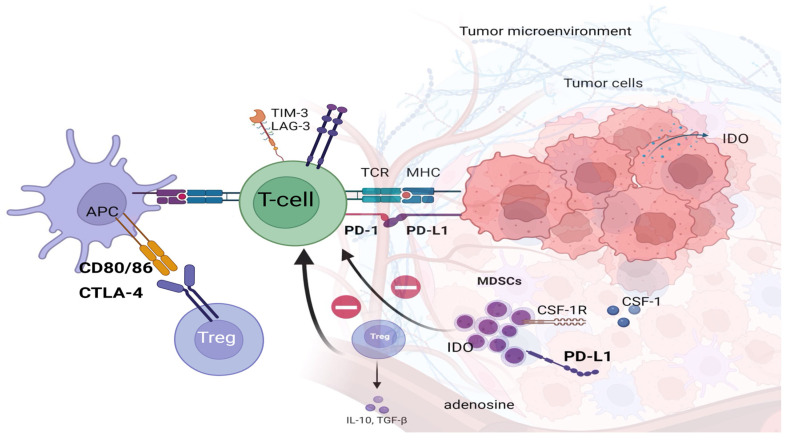
The figure presents cells in the tumor microenvironment expressing PD-L1 and interactions between tumor cells and immune system cells.

The binding of PD-1 to PD-L1 or PD-L2 in host tissues suppresses T-cell receptor (TCR) signaling and CD28 co-stimulation, thus restricting T-cell interactions with target cells [25,26]. Consequently, this process results in T-cell inactivation and loss of their ability to proliferate [27,28,29,30]. Figure 1 presents the interactions between tumor cells and the immune system. Table 1 presents the definitions of the primary and secondary resistance to anti-PD1 therapy, while Table 2 presents the main mechanisms of primary and secondary resistance. An overview of the alterations present in specific cells leading to resistance to anti-PD1 therapy is shown in Table 3.

**Table 1:** Definitions of anti-PD1 therapy resistance—consensus by Kluger et al. [31].

Resistance Phenotype	Drug Exposure Requirement	Best Response	Confirmatory Scan for PD Requirement	Confirmatory Scan Time Frame	Neoadjuvant Therapy Resistance	Adjuvant Therapy Resistance
**Primary resistance**	≥6 weeks	PD; SD for <6 months *	Yes *	≥4 weeks after initial disease progression **	major pathological response before the surgery + requirements for primary resistance	Timing of last dose prior to PD <12 weeks + confirmatory biopsy ***
**Secondary Resistance**	≥6 months	CR, PR, SD for >6 months *	Yes *	≥4 weeks after disease progression **	no major pathological response before the surgery + requirements for secondary resistance	Timing of last dose prior to PD ≥12 weeks + confirmatory biopsy ***

PD, SD, PR, CR- response per RECIST; * Indolent tumor types might require modification of the time frame. ** Other than when tumor growth is rapid and patients are deteriorating clinically. *** Confirmatory biopsy replaces confirmatory scan.

## 3. Primary Resistance Mechanisms

In clinical practice, primary resistance is typically defined as tumor progression observed at the initial evaluation following therapy initiation, usually around week 12. This type of resistance is seen in many patients, ranging from 40% to 65% [12,32,33], depending on whether the therapy is administered as first-line treatment or following progression under other systemic treatments [9,34,35]. Primary resistance mechanisms include (a) resistance of the IFN-γ -signaling pathway [7,36,37]; (b) insufficient antigen presentation and/or antigen recognition [37,38,39,40,41]; (c) immunosuppressive tumor microenvironment [42,43,44,45,46,47,48,49]; (d) intrinsic oncogenic pathway [50].

### 3.1. Interferon Gamma Signaling Pathway

Interferons are some of the key proteins involved in generating an immune response against cancer. IFN-γ is produced and interacts with the IFN-γ receptor upon T-cell recognition of tumor antigens. Under normal conditions, this interaction activates Janus kinase (JAK1/2), leading to the phosphorylation of signal transducers and transcription activators (STAT), which drives the expression of IFN-γ-stimulated genes. Diminished sensitivity to the interferon signaling pathway appears to be a significant way cancer cells resist anti-PD-1 drugs, as evidenced by a study by Zaretsky et al. (2016) [37].

IFN-γ signaling may be attenuated through various mechanisms; however, a primary mechanism identified in melanoma is the loss of PTEN expression. PTEN is a tumor suppressor that downregulates the activity of the PI3K signaling pathway in physiological conditions. The PI3K pathway is crucial in promoting cancer cell proliferation and survival. Loss of PTEN, which occurs in 30% of melanomas, leads to hyperactivation of PI3K signaling and a concomitant reduction in interferon γ and granzyme B expression by antigen-specific T cells. The reduction in IFN-γ hinders the mobilization of additional immune cells and disrupts the activation of antiproliferative and pro-apoptotic signaling in tumor cells [51,52,53,54,55]. Suppressing apoptosis-inducing factors such as caspase-8 and TRAIL allows melanoma cells to evade immune cell-mediated death [56,57]. The prevention of immune-mediated apoptosis and destruction in these cells is linked to the overexpression of pro-survival genes such as BCL2 or BCL-XL [58].

IFN-γ exerts its effects via the JAK-STAT1 signaling pathway, and consequently, homozygous loss-of-function mutations in genes encoding JAK1 or JAK2 result in resistance to anti-PD-1 therapy through resistance to IFN-γ-mediated growth inhibition and induction of apoptosis [55]. JAK1/2 mutations decrease T chemotaxis through reduced secretion of CXCL10 and insensitivity to IFN-γ, thereby impairing its capacity to inhibit the proliferation of cancer cells [36,37,48]. STAo IFN-T molecules are oncogenes, but they also upregulate PD-L1 expression [59].

### 3.2. Insufficient Antigen Presentation and/or Recognition

**Figure 2:**
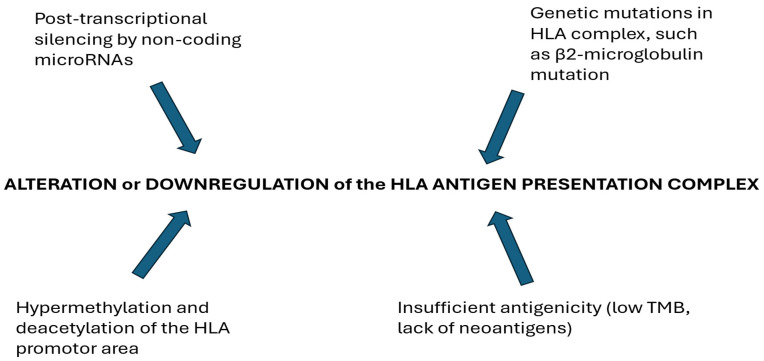
Genetic and non-genetic modifications that may result in alteration or downregulation of the HLA antigen presentation complex.

Antigen presentation in the tumor microenvironment (TME) occurs primarily through the Class I major histocompatibility complex (MHC I) pathway. Tumors can evade T-cell-mediated killing by disrupting MHC class I. Alteration or downregulation of the HLA antigen presentation complex may be caused by a genetic mutation in the complex, such as beta-2-microglobulin, post-transcriptional silencing by non-coding microRNAs, and hypermethylation and deacetylation of the HLA promoter area. A low tumor mutation burden and lack of neoantigens contribute to this resistance [60]. Figure 2 summarizes genetic and non-genetic modifications that may impair antigen presentation. Effective presentation of clonal neoantigens, which results from tumor-specific mutations, is essential for triggering T-cell immunoreactivity and enhancing the efficacy of immune checkpoint blockade. McGranahan et al. demonstrated that tumors with a greater load of clonal neoantigens exhibit better responses to PD-1 inhibitors, underscoring the importance of the effective presentation of neoantigens for the success of immunotherapy [39].

**Figure 3:**
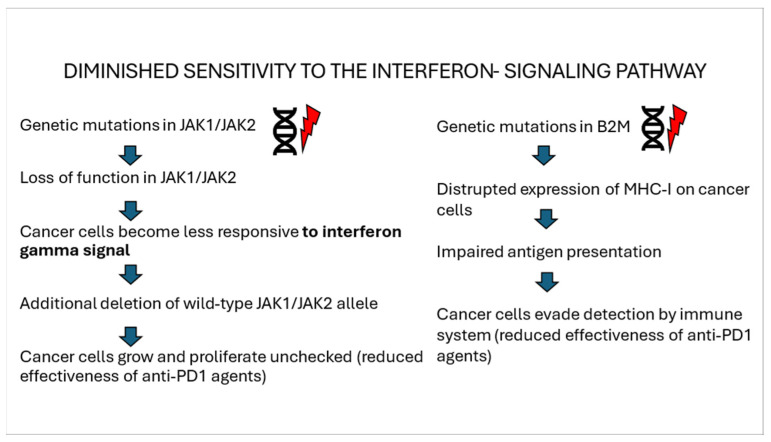
Influence of JAK1/JAK2 and B2M gene mutations on resistance to anti-PD1 treatment.

**Figure 4:**
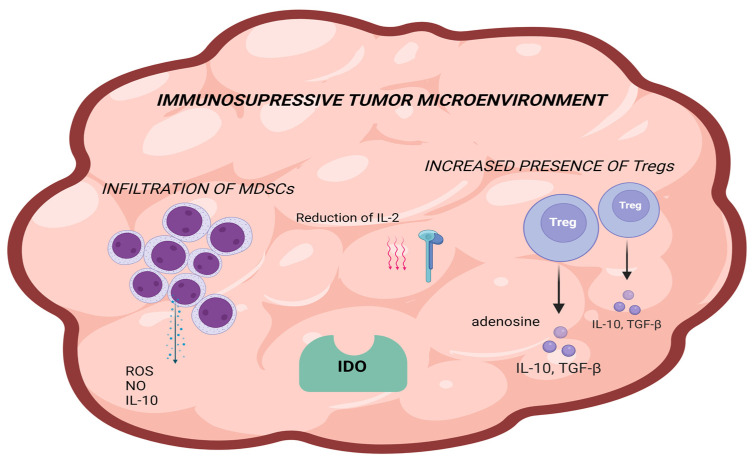
Summary of alterations in tumor cells that create an immunosuppressive microenvironment: infiltration of MDSCs producing ROS, NO, and IL-10; reduction of IL-2; presence of IDO; increased presence of Tregs producing IL-10, TGF-β, and adenosine. Created with BioRender.com.

Beta-2-microglobulin (B2M) plays a critical role in the adequate assembly and stabilization of HLA I [61]. Mutations in B2M disrupt HLA class I function, impairing antigen presentation and reducing T-cell-mediated cytotoxicity. Furthermore, it has been demonstrated that loss of heterozygosity (LOH) at the B2M locus occurred three times more frequently in non-responders than in responders, and loss of both copies of B2M was found only in non-responders among patients with melanoma treated with anti-PD-1 [62]. Hypermethylation of the HLA genes, which results in reduced expression, has also been observed in melanoma resistant to anti-PD1 [63,64,65,66]. A summary of the influence of JAK1/2 and B2M gene mutations on resistance to anti-PD-1 treatment is presented in Figure 3. Figure 4 shows the influence of this mutation, including mutations in JAK1/JAK2, on resistance to anti-PD1 treatment.

A low mutation burden (LMB) is also a significant factor contributing to resistance. The likelihood of T-cell recognition and response decreases when tumors generate fewer neoantigens due to fewer mutations. Multiple studies have shown that insufficient immunogenic targets for T cells in tumors with a lower overall mutation load reduce the effectiveness of anti-PD-1 agents [38,67]. According to a meta-analysis conducted by Ning et al., melanoma patients with a high tumor mutational burden (TMB) experienced over a 50% decrease in the risk of death or disease progression when treated with ICIs. This beneficial effect was exclusively observed in patients undergoing single-agent ICI therapy, such as anti-PD-L1 or anti-CTLA-4. Moreover, individuals with high TMB demonstrated notably better overall survival rates than those with low TMB [68].

### 3.3. Immunosuppressive Tumor Microenvironment

Intrinsic characteristics of tumor cells play a crucial role in primary resistance to ICIs; however, the tumor microenvironment and immune system also significantly influence this resistance [69,70]. Components of the TME, such as regulatory T cells (Tregs), macrophages, dendritic cells, and myeloid-derived suppressor cells (MDSCs), strongly influence immunotherapy outcomes. Elevated levels of tumor-associated macrophages (TAMs), Tregs, and MDSCs in the tumor stroma have been consistently associated with poor prognosis [71,72]. Studies have indicated that patients who respond well to immunotherapy often have higher densities of CD8+T cells within their melanoma tumors before the therapy [73,74]. Favorable responses correlate with immune cells within the cancer, rather than just at the tumor’s margins, and with increased T-cell and macrophage infiltration into the TME after starting treatment [37,50,75,76,77,78,79,80,81].

Alterations in the tumor microenvironment, including the increased presence of immunosuppressive cells, such as regulatory T cells (Tregs) or myeloid-derived suppressor cells (MDSCs), can establish an immunosuppressive environment that dampens the effectiveness of anti-PD1 therapy. Regulatory T cells—Tregs are key players in immunosuppression within the tumor microenvironment, enabling tumors to evade immune detection by suppressing antigen-specific T-cell functions [82]. These immunosuppressive cells inhibit effector T-cell activity directly or indirectly by secreting cytokines such as interleukin 10 (IL-10) and transforming growth factor β (TGF-β). Tregs also produce immunosuppressive factors, including IL-10, TGF-β, and extracellular adenosine, and induce deprivation of IL-2 in TME [82]. Tregs absorb IL-2 in the tumor microenvironment more efficiently than other T and NK cells, depriving them of this critical cytokine [83]. By expressing high levels of CTLA-4, Tregs regulate immune activity by actively reducing the presence of CD80/CD86 (costimulatory molecules on APCs shown in Figure 1) through CTLA-4-mediated processes, such as trogocytosis. This reduction hinders the activation of naïve T cells and elevates free PD-L1 expression on dendritic cells, amplifying their suppressive impact on effector T cells. Moreover, the balance of CD80 and CD86 expression on APCs significantly influences Treg-mediated suppression, with each molecule interacting differently with CD28 and CTLA-4 to modulate immune responses [84,85]. In vivo studies have shown that Tregs, by inducing high levels of PD-1 expression in CD8+T cells, contribute to primary resistance to anti-PD-1 therapy [79].

Macrophages are immune cells responsible for phagocytosis and production of pro-inflammatory cytokines. Tumor-associated macrophages (TAMs) can either promote or suppress tumor growth. Rather than distinct categories, TAMs are now widely recognized to exist along a spectrum of phenotypes ranging from M1-like, which is generally associated with antitumor activity during the initial immune response, to M2-like, which promotes tumor growth [86]. Additionally, TAMs hinder the effectiveness of immunotherapy by preventing the recruitment of CD8+ T cells to the tumor [71,72]. Research has indicated that lower levels of TAMs correlate with improved responses to immunotherapy in melanoma patients [72]. Simultaneously, myeloid-derived suppressor cells (MDSCs) support angiogenesis and cell invasion by secretion of TGF-β and vascular endothelial growth factor (VEGF), helping preserve the tumor immunosuppressive microenvironment. MDSCs produce factors, such as reactive oxygen species (ROS), nitric oxide (NO), and IL-10, which suppress antigen-specific and nonspecific T-cell responses and promote tumor invasion and angiogenesis [87,88]. MDSCs inhibit T cells using nutrients such as arginine and tryptophan [89]. Therefore, MDSC infiltration into tumors is crucial for responding to PD-1/PD-L1 blockade therapy. Studies have shown that targeting MDSCs can improve the efficacy of anti-PD-1 [90,91]. Research has shown that lower MDSC levels are associated with a better response to immunotherapy in patients with melanoma [71,72,92]. Bouhris et al. highlighted the role of immature vessels and endothelial cells and their effect on Tregs and all tumor niches. These cells induce apoptosis of CD8+ T cells, polarize TAMs to an inhibitory phenotype, and secrete VEGF, which directly suppresses CD8+ cells and activates Tregs [93]. An increase in each of these cells might be linked to a worse prognosis in melanoma patients [94]. While they hold potential as predictive markers for immunotherapy, additional studies are necessary to confirm this.

Indoleamine 2,3-dioxygenase (IDO) is an enzyme responsible for breaking down tryptophan into kynurenine, creating an immunosuppressive environment in the TME by suppressing the activity of T cells and dendritic cells and enhancing the functions of Treg and MDSC [95]. IDO is another target shown to manifest resistance to PD-1 blockade therapy by accumulating TME immunosuppressants [96]. Furthermore, the metabolic requirements of cancer cells create a TME that can hinder effective immune cell function. Tumor cells and T cells frequently vie for the essential metabolic substrates, including glucose, cholesterol, and specific amino acids such as arginine and glutamine [97,98,99,100]. The acidic nature of TME, driven by elevated tumor-derived lactate and hypoxia from cancer cell metabolism, impairs T-cell activity, as these cells rely on aerobic glycolysis [98,101,102,103,104,105,106,107]. A study by Johnston et al. demonstrated that acidic pH (typical for TME) may lead to selective suppression of T cells by V-domain immunoglobulin suppressor of T-cell activation (VISTA), which may contribute to anti-PD-1 therapy resistance [108]. Moreover, hypoxia stimulates ATP release into the TME, and its subsequent conversion to adenosine leads to activation of the A2A receptor on effector T cells, further suppressing their functionality [102].

Furthermore, the microbial component of TME involves bacteria that alter immune cell infiltration through Toll-like receptor signaling, affecting the efficacy of checkpoint inhibitors [109,110]. Bifidobacterium and other beneficial bacteria are linked to enhanced dendritic cell activity and better outcomes in melanoma models [111,112,113,114]. In contrast, certain bacteria, such as Fusobacterium nucleatum, have been linked to immune suppression and tumor progression [115,116]. Figure 4 presents a summary of alterations that create an immunosuppressive tumor microenvironment.

### 3.4. Intrinsic Oncogenic Pathway Altering TME

Intrinsic resistance mechanisms caused by specific genetic alterations in melanoma affect the efficacy of PD-1 inhibitors. A mutation in the WNT/β-catenin pathway is associated with one of them. These mutations can result in an immunologically “cold” tumor microenvironment that lacks T-cell infiltration. The WNT/β-catenin pathway inhibits dendritic cell recruitment and is essential for T-cell priming and the start of an efficient immune response against tumors. Therefore, tumor cells that show active WNT/β-catenin signaling often resist immune checkpoint inhibitors [50].

Another critical pathway contributing to resistance is the loss of PTEN function, which was mentioned in the context of the IFN-γ signaling pathway. PTEN is a tumor suppressor gene that downregulates the PI3K/AKT signaling pathway. Loss of PTEN causes hyperactivation of the PI3K/AKT pathway, which is associated with an immunosuppressive tumor microenvironment. PTEN loss explicitly facilitates the attraction of immunosuppressive cell populations, including Tregs and MDSCs, while impeding the infiltration and function of cytotoxic T lymphocytes (CTLs). Consequently, tumors that lack PTEN are frequently unresponsive to anti-PD-1 drugs [51]. Furthermore, PI3K/AKT pathway mutations can autonomously lead to resistance against PD-1 inhibitors. Activation of this pathway can increase the synthesis of immunosuppressive cytokines and growth factors, thus exacerbating inhospitable conditions for immune cells. This signaling cascade promotes the survival and growth of tumor cells while impairing the immune system’s capacity to generate an effective response against the tumor [117].

## 4. Innate Resistance Mechanisms

Recent studies have deepened our understanding of innate resistance to anti-PD-1 in melanoma. The expression of a distinct group of genes termed the innate anti-PD-1 resistance signature (IPRES) is linked to the transformation of melanoma cells into a mesenchymal subtype, which resembles a stem cell-like phenotype [118,119]. This upregulation, possibly driven by inflammation within the tumor microenvironment, enhances tumor plasticity and is associated with resistance to checkpoint inhibitor therapy [119,120]. The IPRES gene set promotes improved tumor plasticity in melanoma, allowing cells to adapt and survive under therapeutic pressure. This plasticity facilitates evasion of immune surveillance and resistance to treatments aimed at reactivating immune responses, such as anti-PD-1 therapies [118,119]. The tumor microenvironment plays a crucial role, as inflammatory signals within this niche can induce the expression of these resistance-associated genes [120]. Furthermore, epithelial-to-mesenchymal transition (EMT) genes play a significant role in resistance mechanisms. Up-regulation of genes related to EMT, such as AXL, TWIST2, WNT5a, LOXL2, ROR2, TAGLN, and FAP, is associated with an increased capacity of melanoma cells to undergo phenotypic changes, enhancing their invasiveness and survival ability. These changes in EMT are often accompanied by up-regulation of pro-inflammatory cytokines like TNF-α, which further supports a resistant and aggressive tumor phenotype [119,121]. These findings highlight the complexity of immune resistance in melanoma and the potential of combination therapies.

## 5. Secondary Resistance Mechanisms

Secondary resistance to anti-PD-1 therapy refers to the phenomenon in which patients who initially respond later experience disease progression. This resistance arises due to several adaptive mechanisms that tumors develop under the selective pressure of immune checkpoint blockade. Most of these mechanisms are similar to those observed in primary nonresponders.

### 5.1. Tumor Intrinsic Mechanisms

#### 5.1.1. Mutations Within the Interferon Signaling Pathway

One primary mechanism underlying secondary resistance to anti-PD-1 therapy involves the downregulation of antigen presentation on the tumor cell surface, which can be mediated by the upregulation of the interferon γ signaling pathway after exposure to checkpoint inhibitors [122,123,124,125]. Resistance can arise through acquired inactivating mutations in critical components of the interferon signaling pathway, such as JAK1 and JAK2 [34,35]. Acquired loss-of-function mutations in JAK1/2 were found in patients with relapsed melanoma tumors with prior pembrolizumab treatment. The mechanisms were similar to the reported ones responsible for primary resistance to therapy involving mutations in this signaling cascade [34]. In these patients, tumor cells did not show activity in the IFNγ signaling pathway, resistance to the antiproliferative effects of IFNγ, and lack of surface expression of PD-L1 and MHC class I in response to IFNγ [35]. This phenomenon highlights how PD-1/PD-L1 blockade can selectively promote the survival of tumor cell clones that acquire mutations, enabling them to resist the inflammatory pressures exerted by anti-tumor T cells.

#### 5.1.2. Mutations in Antigen Presentation Pathway Genes

Mutations in antigen presentation pathways can disrupt antigen/neoantigen presentation, contributing to primary and secondary resistance to PD-1/PD-L1 blockade. Although this disruption may exist at the beginning of the study in cases of primary resistance, it has also been observed during treatment. For example, the loss of β2-microglobulin (β2M), a protein necessary for the surface expression of MHC class I molecules, has been reported following therapies such as adoptive cell therapy or IL-2 treatment [120]. The loss of β2M results in impaired T-cell recognition of tumor cells. Similarly, clinical cases of acquired resistance to PD-1 blockade in melanoma have demonstrated homozygous truncating mutations in β2M that were absent in the baseline tumor but emerged during treatment [35]. This mutation-induced escape from T-cell detection highlights how immunotherapies such as PD-1/PD-L1 blockade can exert selective pressure on tumor cells. Clonal expansion of these cells leads to relapse in patients who initially responded to treatment.

Inflammatory stress has been found to drive melanoma cell dedifferentiation towards a neural crest lineage, characterized by upregulation of nerve growth factor receptor (NGFR) and downregulation of melanosomal antigens, such as MART-1 [121,122,123]. A similar pattern has been observed with mutational neoantigens, critical for T-cell-mediated tumor recognition during anti-PD1 therapy [124,125]. Recent studies in non-small cell lung cancer (NSCLC) have shown that patients who developed acquired resistance to PD-1/PD-L1 inhibitors experienced a loss of several mutation-associated neoantigens during treatment. The loss of these neoantigens was found to trigger clonal T-cell expansion in vitro, showing their ability to stimulate a T-cell response [126]. This highlights the need to understand better how the neoantigen landscape changes in patients who develop acquired resistance to PD-1/PD-L1 therapy.

#### 5.1.3. Loss of Tumor Suppressor Genes

The loss of tumor suppressor genes such as PTEN significantly contributes to primary and acquired resistance to PD-1/PD-L1 blockade. As mentioned above, loss of PTEN activates the PI3K-AKT pathway, promoting tumor cell survival and proliferation and reducing T-cell infiltration in the tumor microenvironment. Analysis of data from the Cancer Genome Atlas (TCGA) in melanoma has shown that PTEN deletions and loss-of-function mutations are more frequent in non-T-cell-inflamed tumors compared to T-cell-inflamed tissues, which correlates with poor responses to immunotherapy [50,127,128]. Furthermore, mouse melanoma models have shown that PTEN loss reduces T-cell infiltration and increases the expression of inhibitory cytokines and autophagy, supported by clinical studies in metastatic melanoma [128]. These changes allow tumor cells to escape T-cell-mediated killing, further promoting resistance.

### 5.2. Tumor-Extrinsic Mechanisms

#### 5.2.1. T-Cell Exhaustion and Memory T Cells

A key tumor-extrinsic mechanism that contributes to secondary resistance is T-cell exhaustion. T cells in the tumor microenvironment experience chronic antigen stimulation, leading to functional exhaustion characterized by the upregulation of multiple co-inhibitory receptors, including PD-1, TIM-3, TIGIT, and LAG-3. T-cell exhaustion is a significant factor in the failure of anti-PD-1 therapies, as demonstrated in clinical and preclinical studies [129]. Although anti-PD-1 blockade can initially reinvigorate exhausted CD8+ T cells (TEX), the effect is often temporary due to the underlying epigenetic programs that maintain the exhausted state, limiting the therapy’s long-term efficacy [75]. Recent studies suggest that the TOX high mobility group box protein associated with thymocyte selection is a crucial driver of T-cell exhaustion, reinforcing the persistence of the exhausted phenotype despite anti-PD-1 therapy [75,130]. Tumor-infiltrating TEX cells progress through different epigenetic states, initially showing some plasticity, which allows functional restoration. However, over time, these cells enter a fixed state of epigenetic dysfunction, where chromatin becomes inaccessible, making the cells resistant to further reinvigoration by blocking PD-1 [131]. The progression to that irreversible state is associated with poor therapeutic outcomes and relapse [75]. The ratio of reinvigorated TEX cells to tumor burden at treatment has been linked to the amount of clinical response to anti-PD-1 treatment [79]. If the tumor burden remains high and TEX fails to eliminate it, these cells become exhausted, locking them into a fixed dysfunctional epigenetic state unresponsive to further PD-1 blockade [75]. One of the potential strategies to overcome anti-PD-1 resistance is addressing CD8+ T-cell exhaustion through epigenetic and metabolic manipulation. Hypoxia within the tumor microenvironment drives T-cell exhaustion by activating hypoxia-inducible factors (HIFs), such as HIF-1α, which epigenetically suppress cytotoxic cytokines (e.g., IFN-γ, TNF-α). Strategies like inhibiting HIF-1α with PX478 or enhancing histone demethylation via KDM6B can reprogram exhausted T cells and restore antitumor activity [126].

Another factor contributing to resistance is the limited involvement of memory T cells (TMEM) in response to PD-1 therapy. PD-1 blockade predominantly activates transcriptional programs in effector T cells (TEFF), which have lower proliferative capacity and shorter lifespans than TMEM. In contrast, TMEM cells are essential for long-term immune responses, including sustained anti-tumor activity [132]. A lack of TMEM, combined with the exhaustion of effector T cells (TEX), often leads to tumor progression after an initial response to therapy. This underscores the need for epigenetic reprogramming strategies to restore TEX function and promote memory cell involvement [75].

Despite limited circulating TMEM activation, anti-PD-1 therapy demonstrates significant benefits in neoadjuvant settings. Studies in esophageal squamous cell carcinoma (ESCC) mouse models demonstrated enhanced CD8+ tissue-resident memory T-cell (TRM) infiltration in the tumor microenvironment. These TRMs persisted long-term and retained antitumor activity even upon re-exposure to carcinogens, reducing tumor recurrence. These findings emphasize the therapy’s potential to reshape the immune microenvironment and open avenues for early-stage cancer treatment strategies [127].

#### 5.2.2. Immunosuppressive Microenvironment

The tumor microenvironment in melanoma can become increasingly immunosuppressive with time, contributing to resistance. As with primary resistance mechanisms, secondary resistance is linked to changes in the balance between immune effector cells and suppressor cells within the tumor microenvironment. Regulatory T cells, myeloid-derived suppressor cells (MDSCs), and tumor-associated macrophages secrete immunosuppressive cytokines such as TGF-β and IL-10, which inhibit T-cell activity and promote an environment that favors tumor growth. This secretion reduces the effectiveness of anti-PD-1 therapy, allowing the tumor to escape immune control [133]. PD-1/PD-L1 inhibition is also associated with upregulating other inhibitory pathways, such as EZH2, LAG-3, TIM-3, TIGIT, and VISTA, silencing immune activity [52,134,135,136].

#### 5.2.3. Metabolic Competition

Tumor cells often outcompete T cells for essential metabolic substrates such as glucose, cholesterol, arginine, and glutamine within the microenvironment [92,93,94,95]. This metabolic competition affects T-cell function and limits their ability to respond effectively to anti-PD-1 therapy [95,137]. The expression of inflammatory signaling molecules like TGF-β, indoleamine-2,3-dioxygenase (IDO), IL-10, and arginase by stromal cells and leukocytes within the tumor microenvironment contributes to the suppression of immune cell activity [138,139,140,141,142]. IDO expression by melanoma cells catabolizes tryptophan, resulting in impaired function of dendritic cells, suppressed proliferation of T cells, and increased MDSCs and Treg infiltration, further contributing to immune evasion [143]. Acidic conditions within the tumor microenvironment, caused by increased lactate production and hypoxia, further alter T-cell activity, as they depend on aerobic glycolysis for optimal function [93,96,97,98,99,100,101,102]. In contrast, Tregs are less dependent on these substrates, allowing them to thrive in this metabolically stressed environment, thus improving overall immunosuppression [144].

## 6. Management of Patients with Melanoma Resistant to Anti-PD1 Therapy

The treatment of patients with melanoma resistant to anti-PD-1 therapy remains a significant challenge, as highlighted in various studies presented at ESMO 2024. Several combinations of therapeutics with mixed outcomes have been explored in randomized phase II and III trials. The SWOG-S1616 trial, involving anti-PD-1-resistant melanoma patients, demonstrated that combining ipilimumab and nivolumab (higher ORR compared to ipilimumab monotherapy, HR 0.63, indicating an improvement in PFS) [128]. Other studies, such as NCT02773179, also showed an improvement in ORR when combining ipilimumab with nivolumab in patients resistant to PD-1 [129].

Furthermore, single-arm trials like RELATIVITY-047 and DREAMseq explored novel combinations, including LAG-3 and BRAF + MEK inhibitors for BRAF-mutant melanoma, showing encouraging response rates. However, there is still a lack of phase III data to compare the effectiveness of these options across large patient populations. The phase I study (NCT03005782) combining fianlimab (anti-LAG-3) and cemiplimab in advanced melanoma, including patients with previous neo/adjuvant treatment (phase III study combining fianlimab with cemiplimab in naïve metastatic melanoma pts-NCT05352672), shows promise in inducing durable responses [130]. Patel et al. accessed the safety and efficacy of the first-in-class CXCR1/2 inhibitor SX-682 in combination with pembrolizumab in patients with metastatic melanoma with disease progression on anti–PD–1 therapy [131]. In the phase II trial by Weiss et al., sotigalimab (CD40 agonist antibody) combined with nivolumab demonstrated a safety profile consistent with the known toxicities of each agent. Notably, the combination therapy resulted in durable and prolonged responses in patients with melanoma resistant to anti-PD-1 therapy. These findings underscore the potential of combining immune-stimulatory agents with checkpoint inhibitors to overcome resistance and enhance clinical outcomes [132].

Lipo-MERIT trial (NCT02410733), a phase I study investigating the use of personalized mRNA vaccines (BNT111) targeting four specific neoantigens (NY-ESO-1, MAGE-A3, tyrosinase, and TPTE) in melanoma patients, aiming to stimulate a potent immune response by training T cells to recognize and destroy tumor cells expressing these mutations, with promising results observed when combined with checkpoint inhibitors [133]. BioNTech’s phase 2 trial (NCT04526899) showed positive results, with BNT111 plus cemiplimab significantly improving the objective response rate (ORR) in patients with anti-PD-1-refractory/relapsed, unresectable stage III/IV melanoma.

In a phase 1/2 trial (NCT03047928), nivolumab combined with a PD-L1/IDO peptide vaccine demonstrated promising efficacy and survival benefits for patients with metastatic melanoma. These findings were highlighted during the 2022 AACR Annual Meeting, showcasing the potential of integrating immunotherapy with peptide vaccines to improve treatment outcomes in advanced melanoma cases.

Table 4 presents therapeutic options after progression on anti-PD1 treatment in patients with melanoma.

## 7. Emerging Insights and Potential Therapeutic Strategies

Recent studies have highlighted the significant role of microRNAs, particularly miR-146a, in modulating immune responses within the microenvironment of melanoma. miR-146a is a crucial regulator of several pro-inflammatory pathways, including the TRAF6/TNF axis in T cells and the JAK/STAT/MHC axis in antigen-presenting cells. Research by Justin et al. demonstrates that miR-146a influences melanoma cell migration, proliferation, mitochondrial fitness, and PD-L1 expression. Combining miR-146a, antagomir, and anti-PD-1 therapy significantly increased the survival rate of melanoma mice compared to using anti-PD-1 therapy alone. These results suggest that targeting miR-146a and blocking PD-1 can increase anti-tumor immune responses, providing a promising way to get around melanoma cells resistant to checkpoint therapy [142].

Recent evidence suggests that targeting myeloid-derived suppressor cells (MDSCs) and regulatory T cells along with PD-1 inhibitors could potentially improve the efficacy of anti-PD-1 therapies. For example, blocking the CXCR2 receptor, which is involved in attracting MDSCs to the tumor location, has been demonstrated to enhance the efficacy of anti-PD-1 therapy in preclinical models of melanoma [90].

Another promising approach involves modulating metabolic pathways within the tumor and immune cells. Tumors frequently exploit these pathways to create an immunosuppressive microenvironment. For example, melanoma cells can increase the enzyme indoleamine 2,3-dioxygenase (IDO) production, which reduces the amount of tryptophan in the tumor microenvironment, suppressing T-cell function. Early-phase clinical trials investigating IDO inhibitors combined with anti-PD-1 therapy showed promise [128]. A Phase II clinical trial demonstrated that combining the IDO pathway inhibitor indoximod with pembrolizumab was well-tolerated and yielded a 51% objective response rate, including a 20% complete response rate and a 70% disease control rate, suggesting significant antitumor efficacy in advanced melanoma, particularly in PD-L1-positive patients [143]. Although early-phase trials suggested promise for combining IDO inhibitors with anti-PD-1 therapy, the lack of efficacy observed in a Phase III trial with epacadostat and pembrolizumab has led to scepticism regarding this approach [144,145].

Moreover, targeting tumor mutational burden (TMB) is another potential strategy. High TMB is associated with producing neoantigens, which can enhance tumor immunogenicity and improve response to anti-PD-1 therapy. However, resistance to treatment can occur through various mechanisms, such as reduced neoantigen expression due to immune editing or loss of heterozygosity at the HLA locus, resulting in diminished T-cell recognition. Strategies to increase TMB or improve the presentation of neoantigens are being explored, including personalized neoantigen vaccines, as methods to overcome resistance to anti-PD-1 therapies in melanoma [36].

A study by Gopalakrishnan et al. suggests that the gut microbiome influences responses to anti-PD-1 immunotherapy in melanoma patients. A “favorable” microbiome enhances immune responses, while an “unfavorable” microbiome impairs them, highlighting the potential of microbiome modulation in overcoming immunotherapy resistance [146].

Advances in understanding epigenetic regulation have also opened new possibilities for overcoming resistance to PD-1 blockade. Epigenetic modifications, such as DNA methylation and histone modification, can lead to the silencing of genes crucial for immune responses. Agents targeting epigenetic regulators, such as histone deacetylase (HDAC) inhibitors, are currently being investigated with anti-PD-1 therapy to reverse immune evasion mechanisms and improve anti-tumor immune responses. Although the focus of these strategies is currently on lung cancer, there is also the possibility of applying these therapeutic options to the treatment of melanoma [147].

GDF-15 (growth differentiation factor 15) is a stress-induced cytokine known to suppress immune responses and contribute to tumor immune evasion. In preclinical trials, blocking GDF-15 significantly enhanced the effectiveness of anti-PD-1 checkpoint inhibitors. A first-in-human trial (GDFATHER-1/2a, NCT04725474) evaluated the anti-GDF-15 antibody visugromab in combination with nivolumab in patients with advanced cancers resistant to anti-PD-1/PD-L1 therapies. Promising results included durable responses in non-squamous non-small cell lung cancer and urothelial cancer. The treatment increased tumor infiltration and activity of cytotoxic T cells, suggesting GDF-15 inhibition could help overcome resistance to immune checkpoint inhibitors [148]. These findings and other emerging therapeutic approaches, including those tested in NSCLC and other cancers, offer hope for overcoming resistance to immune checkpoint inhibitors, potentially expanding effective treatment options for melanoma and other challenging malignancies. Table 5 presents a summary of genetic drivers of resistance to immunotherapy found in recent studies.

**Table 5:** Summary of genetic drivers of resistance to immunotherapy that recently have been found.

Gene	Immune Influence	Study
**EZH2**	Affects antigen presentation and T-cell infiltration.	[149]
**HDAC6**	Increases IL-10 and PD-L1, suppressing the immune response.	[150]
**RASSF5 & ITGB2**	Impairs immunogenicity generation.	[150]
**KDM5B**	Recruits SETDB1 and modifies immune gene expression.	[151]
**SETDB1**	Regulates immune-related gene clusters and antigen presentation.	[147,152,153]
**FTO**	Elevates PD-1 expression via autophagy.	[154]
**LAG3**	Negatively regulates T cells by binding to MHC II.	[155,156,157]
**STING**	Modulates metabolism, MHC I, and PD-L1 expression.	[158]
**NLRP3**	Influences inflammatory signals and macrophage activity.	[159,160,161]
**PAI-1**	Affects macrophage polarisation, autophagy cycle	[162,163,164]

## 8. Conclusions

Immunotherapy, particularly anti-PD-1 agents, has significantly revolutionized cancer treatment. Although solid tumors can be treated effectively, only a subset of patients show an initial response to treatment, and even fewer achieve long-term benefits and survival due to primary and secondary resistance mechanisms. Therefore, examining the resistance mechanisms to anti-PD1/PDL1 therapies is crucial to developing strategies to overcome this challenge. The diversity in patient resistance mechanisms highlights the importance of personalized treatment strategies. The ongoing search for reliable biomarkers to predict response or resistance to anti-PD1 therapy is critical to optimizing these strategies. To overcome resistance to anti-PD1 in melanoma, a deeper understanding of the underlying mechanisms and developing novel therapeutic approaches will be required. Combining anti-PD1 therapy with other treatment modalities, such as targeted therapies, vaccines, or additional immune checkpoint inhibitors, promises to improve patient outcomes and overcome resistance.

## Figures and Tables

**Table 2:** Identified alterations leading to primary and secondary resistance mechanisms.

Primary Resistance—Main Mechanisms	Alterations Leading to Resistance
Resistance of IFN-γ-signaling pathway	- mutations in JAK1/2 - loss of PTEN expression
Insufficient antigen presentation and/or antigen recognition	- genetic mutation in HLA complex (beta-2 microglobulin) - post-transcriptional silencing by non-coding microRNAs - hypermethylation and deacetylation of the HLA promoter area - low tumor mutation burden - lack of neoantigens
Immunosuppressive tumor microenvironment	- increased presence of immunosuppressive cells (Tregs, MDSCs) - elevated levels of TAMs - elevated IDO - presence of Fusobacterium nucleatum
Intrinsic oncogenic pathway	- mutation in the WNT/β-catenin pathway - loss of PTEN
**Secondary Resistance—Main Mechanisms**	**Alterations Leading to Resistance**
Mutations within IFN-γ-signaling pathway	- acquired loss of function JAK1/2 mutation
Mutations in antigen presentation pathway	- loss of β2-microglobulin - loss of mutation-associated neoantigens - upregulation of NGFR, downregulation of MART-1
Loss of tumor suppressor genes	- loss of PTEN
T-cell exhaustion and memory T cells	- upregulation of multiple co-inhibitory receptors (PD-1, TIM-3, TIGIT, LAG-3) - lack of TMEM
Immunosuppressive microenvironment	- increased presence of immunosuppressive cells (Tregs, MDSCs) - upregulating inhibitory pathways (EZH2, LAG-3, TIM-3, TIGIT, VISTA)
Metabolic component	- expression of IDO, IL-10, arginase, TGF-β

**Table 3:** Summary of alterations in specific cells leading to resistance to anti-PD1 therapy.

Cell	Observed Alteration Leading to Resistance
Tumor cell	Mutations in IFN-γ signaling pathway: - Loss of PTEN - Loss of function mutations in JAK1/2
Tumor cell	Mutations in HLA antigen presentation complex: - beta-2-microglobulin complex - posttranscriptional by non-coding microRNA - hypermethylation/deacetylation of the HLA promotor area
Tumor cell	Low mutation burden, lack of neoantigens
Tregs	- secretion of IL-10, TGF-β, extracellular adenosine - absorption of IL-2 - reduction of CD80/86 presence (through CTLA-4 mediated process)
MDSCs	Promotion of angiogenesis and tumor invasion: - secretion of TGF-β and VEGF - secretion of ROS, NO, IL-10 Suppression of T-cell response: - arginine, tryptophan - secretion of ROS, NO, IL-10
TAMs	Prevention of recruitment of CD8+ T cells
Tumor cell	Mutation in WNT/β-catenin
Tumor cell	Expression of IPRES (EMT): - upregulation of genes such as AXL, TWIST2, WNT5a, LOXL2, ROR2, TAGLN, and FAP
CD8+ T-cell	Exhaustion (TEX): - upregulation of coinhibitory receptors: PD-1, TIM-3, TIGIT, LAG-3

**Table 4:** A summary of nonclinical and clinical trials of therapeutic options after progression on anti-PD-1 treatment in patients with melanoma.

Management of Anti-Pd1 Therapy Resistance			
Nonclinical Trial Options (Approved By FDA)		Clinical Trials Options	
Treatment	Response Rate		
Ipilimumab 1 mg/kg + pembrolizumab [134] Ipilimumab 3 mg/kg + nivolumab [135]	29% 29%	BNT111 + cemiplimab Phase II	(NCT04526899)
Nivolumab + relatlimab [136,137]	11%	Avelumab (anti-PDL1) Phase I	NCT01772004)-completed
BRAF/MEK inhibitors for BRAF-mutated melanoma [138]	48%	CXCR1/2 inhibitor SX-682 + pembrolizumab Phase I	(NCT03161431)
Tumor-infiltrating lymphocytes [139,140]	49%	Nivolumab + PD-L1/IDO peptide vaccine Phase I/II	(NCT03047928)
Chemotherapy [141]	22%	Sotigalimab + nivolumab Phase II	(NCT03123783)
		BNT111 Phase I	(NCT02410733)
		ONCOS-102 +/− balstilimab Phase II	(NCT0556149)
		TransCon IL-2 β/γ monotherapy/ in combination Phase I/II	(NCT05081609)
		PF-07329640 monotherapy/ in combination bevacizumab or sasanlimab Phase I	(NCT06448364)
		Fianlimab + cemiplimab Phase I	(NCT03005782)

## Data Availability

Not applicable
